# The Book World of Medicine and Science

**Published:** 1897-10-16

**Authors:** 


### THE BOOK WORLD OF MEDICINE AND SCIENCE.

Elements of Human Physiology. By Ernest H. Starling, M.D.Lond., F.R.C.P. Third Edition. (London: J. and A. Churchill. 1897. Price 7s. 6d.)

When a third edition of a book of this character is demanded within so short a time as has been the case in this instance the work of the reviewer is almost reduced to an enumeration of the points in which the present edition differs from its predecessors. Nevertheless, we would not like to let this opportunity pass by without adding our word of praise to the general approval which this book has met with. Its characteristic feature is that it is not redundant, and yet contains all that it is necessary for the student to know on the subjects with which it deals, that is to say, on the principles of physiology, leaving out on the one side all that appertains to the structure of the body, which, whether it be the coarser anatomy or the finer histology, is taught in other text-books; and, on the other side, omitting those practical details which can only be acquired in a laboratory course. With this limitation, we find within the 468 pages of this work an admirable exposition of what it is essential that the student should know of physiology, a key by aid of which he may not only pass examinations but make plain his course in those more purely medical studies in which he will engage so soon as he enters the wards of his hospital. In regard to the present edition some new illustrations have been inserted, but the chief changes that have been made in the book itself are in the sections devoted to the coagulation of the blood and to the central nervous system. It is needless to say that both these subjects are brought up to the level of our most recent knowledge, and that the student who carefully reads what is written regarding them will find himself abreast of to-day so far as the information goes. At the same time, especially in regard to the nervous system, one cannot avoid a little regret that some sort of sketch has not been given of the views which are now passing into the limbo of the past. It seems a pity that the teaching of the students of to-day should be so limited to the present view—to what is actually accepted at the moment—as to leave a great gulf between them and those educated only a dozen years ago. Perhaps for examination purposes it is necessary to stick to the point, but it seems a pity. As a crisp review of the modern position, however, this is an admirable handbook.

Lippincott's Medical Dictionary. Prepared on the basis of Thomas's "Complete Medical Dictionary" by Ryland W. Greene, A.B., with the editorial collaboration of John Ashhurst, Jun., M.D., LL.D., George Piersol, M.D., and Joseph P. Remington, Ph.M., F.C.S. (London: J. B. Lippincott. 1897. 31s. 6d.)

The habit of using a dictionary, like that of solving doubts in an encyclopædia, is a good one, tending as it does to exactitude, and anyone who turns over the pages and notes the great number of the words which are arranged and defined in this book must feel convinced of the necessity of having on his shelves such a work of reference. No doubt it contains many words which are, like many stories, curious if true, but these are the very words which one is apt to come across in unexpected places, and which puzzle one mightily if no explanation of them is forthcoming. We have carefully read through many pages, and it seems to us that the definitions are very well done. It is always a question how far it is worth while giving formulæ to indicate the pronunciation. On the whole, we think it is worth while, as tending to prevent the development of local or even of national variations. We need hardly insist, however, on the importance of studying very carefully the exact meaning of the marks attached to the various letters in these formulæ before building up one's pronunciation of the words themselves. The book appears quite reliable, and is one, we think, that will be found of much value.

Clinical Methods: A Guide to the Practical Study of Medicine. By Robert Hutchinson, M.D., M.R.C.P., and Harry Rainy, M.A., F.R.C.P.Edin., F.R.S.E. (London: Cassell and Co. 1897. Price 9s.)

There are many no doubt who would not hesitate to say that of students' guides to medicine there are already more than enough. It has, however, to be borne in mind how constant is the progress of medical science, how frequent are the changes in its every branch, and that with new knowledge new books are an absolute necessity to the student. In no department of medicine is change more evident than in the methods adopted for the diagnosis of disease, and we think that not only will this book be much used by students but that it will be welcomed by many practitioners who wish to keep themselves abreast of the times. After dealing with case-taking and general condition and appearances, a series of chapters are given treating of the methods adopted in investigating the different regions of the body and the secretions of various organs. The clinical examination of children, the examination of pathological fluids, and clinical bacteriology each have chapters of their own. We may safely say that without exception all the subjects are fully and carefully dealt with, and that what is said on each is thoroughly reliable. At the same time, it is a disappointing and unsatisfying book. This is due to the authors having stuck so strictly to their subject. Within its own limits the book is admirable, but comparatively little attention is given to the meaning of many of the signs elicited, which makes it anything but interesting. The index is very unsatisfactory.

Masters of Medicine. Edited by Ernest Hart, D.C.L. John Hunter. By Stephen Paget. With introduction by Sir James Paget. (London: T. Fisher Unwin. 1897. Price 3s. 6d.)

It has been a happy inspiration that has led to the publication of a series of biographies dealing with men who may fairly be spoken of as "masters in medicine," and if the succeeding volumes fulfil the expectations raised by this life of John Hunter, we shall have in a most handy and readable form the histories of a group of men who have had an unspeakable influence on the current of human progress. In the writing of a biography much depends on the standpoint from which the man and his surroundings are regarded. When in the fulness of time we look back upon past events it is not difficult to point out errors in the best of men. This, however, is criticism, not biography. To take us back, to raise a picture of the past, to make us live in the times and feel the feelings of the men described is what the biographer has to do; this is the task which Stephen Paget has set himself in writing the life of John Hunter, and in this he has achieved a degree of success which whets the appetite for further volumes of the series. It is curious how largely Hunter tells his own story in his letters, those to Jenner being especially full of interest and showing his constant desire for knowledge of apparently small things. But into these we will not enter. It is far too large a subject. All we can do here is to recommend the book, which is most pleasant digging in the garden of the past.

